# The importance of being animate: Information selection as a function of dynamic human-environment interactions

**DOI:** 10.3389/fpsyg.2022.923808

**Published:** 2022-08-23

**Authors:** Rachel L. Bailey, Annie Lang

**Affiliations:** ^1^School of Communication, Florida State University, Tallahassee, FL, United States; ^2^The Media School, Indiana University, Bloomington, IN, United States

**Keywords:** motivational relevance, information selection, encoding, motivated attention, ecological perception, representation-hungry, animacy

## Abstract

This study examined whether the stability of highly relevant animate and inanimate information predicted encoding. Participants (*N* = 149 young adults) viewed audiovisual media and completed a change detection task of screenshots taken from the viewing session. The screenshots were either left as originally viewed or a factor was altered. The factors were all motivationally (relevant to biological imperatives) and story (relevant to the ongoing narrative) relevant. Half were part of an animal and half were part of other environmental information. This was crossed with whether the information was stable or fleeting in the scene (e.g., a person’s clothing vs. their gestures). Changes to animals were more recognized than inanimate information. Changes to fleeting inanimate information were better recognized than changes to stable inanimate information. These findings indicate potential for relevant change in environmental threat and opportunity is adaptively significant and likely to increase attention and encoding across animate and inanimate categories of information.

## Introduction

The motivated attention theoretical framework proposes that limited-capacity attentional resource allocation is biased toward biologically consequent information, or information related to survival and passage of genetic material (Lang et al., 1997; Bradley et al., 2001). Theorists have proposed organisms have evolved two independent motivational systems that organize behaviors to support biological drives, the appetitive and the aversive (Lang et al., 1997; Cacioppo and Gardner, 1999; Norris et al., 2010). The appetitive system automatically supports approach behaviors (e.g., allocating attentional resources and seeking opportunities), and the aversive system automatically supports avoidance behaviors to protect individuals from potential threats. System activation is thought to be directly related to motivationally relevant stimulus proximity, with more proximate information being more activating and behaviorally consequential (Fanselow, 1994; Bradley et al., 2001). In general, this body of work has supported that individuals encode more information when encountering appetitive stimuli (e.g., food, sexual opportunity information) as arousing content increases (e.g., Lang and Yegiyan, 2009; Lang et al., 2013). However, when individuals encounter aversive stimuli (e.g., threats, noxious stimuli), they progress through stages beginning with passive attention, threat identification (greatest encoding), and finally defensive responses as stimuli become more threatening (attentional resources diverted from encoding) (Bradley et al., 2001; Lang and Yegiyan, 2009; Lang et al., 2013).

Other functional approaches to cognition also posit that visual attention and encoding are predisposed *via* selection pressures toward the most salient environmental components. Animacy has been suggested as a key salience factor (e.g., Nairne et al., 2013; Altman et al., 2016), as animals can change their status and affordances quickly and thus require fast initial detection but also consistent monitoring for change (New et al., 2007). This contention has been supported across studies multiple types of animacy including animals and other humans that have animacy potential (New et al., 2007; Calvillo and Jackson, 2014; Altman et al., 2016), words that describe animate concepts (Nairne et al., 2013; Bugaiska et al., 2019), and otherwise inanimate environmental information that is currently moving (Lang et al., 2002; Schurgin and Flombaum, 2017).

Thus, motivationally relevant information about animals and other humans are among the categories of information that have been important for attention and encoding across human evolutionary history. But dynamical accounts of attention and encoding may add important nuance to this perspective. Namely, it may be that some types of motivationally relevant information about animals are more likely to be encoded for later use while other types are left in the environment to coordinate behavior without expending the cognitive resources necessary for encoding. This brief report unites theory from the motivated attention and animate monitoring bias perspectives with work based in ecological perception theory (Gibson, 1977, 1986) and views on representation-hungriness (Clark and Toribio, 1994) to predict what types of information will be better encoded.

## Representation-hungriness

Representations are information we encode from our experiences and thoughts, which can be recalled and acted upon. In the past few decades, the discussion surrounding the existence and physical instantiation of representations has been robust. Some still rely on early accounts of cognition: brains store information in combinatorial bits that are recalled and operated upon in computer-like architecture (e.g., Newell and Simon, 1972; Fodor and Pylyshyn, 1988). Some hold representations need not exist at all aligning with dynamical, complex systems accounts, while others have indicated that without this storage architecture, abstract thought and the ability to plan for the future and remember the past would be impossible (see Chemero, 2000).

A middle-ground position in this debate proposes that our cognitive architecture is very flexible (Clark and Toribio, 1994; Clark and Chalmers, 1998; Chemero, 2000; Clark, 2008) and that humans expend resources to create representations only when encoding information is adaptively useful. Clark and Toribio (1994) called this type of information “representation-hungry.” They argued that representation-hungry information is found in situations that either first, do not require external stimuli to guide behavior, and/or second, have complex parameters to which humans are selectively sensitive and must perform some sort of pattern extraction to complete appropriate behavior (Clark and Toribio, 1994). With this concept of representation-hungriness in mind, this brief report argues that motivationally relevant information, broadly defined as information important for meeting biological imperatives, contains more and less representation-hungry components. Though motivationally relevant information is more likely to be attended to, not all forms of motivationally relevant information are representation-hungry. To understand which may be, we turn to ecological perception theory.

## Complex parameters to which humans are selectively sensitive

From an ecological perception perspective (Gibson, 1977, 1986), the relative importance of information is in what necessary behaviors are afforded by environmental elements—called an affordance. However, even if environmental elements afford needed behaviors they may not be automatically encoded. Most human behaviors merely require that individuals use their environments as scaffolds on which to perform goal-oriented tasks without expending resources on encoding. In representation-hungry situations, individuals may devote resources to extracting complex and potentially abstract information and acting upon it, especially if it affords necessary behaviors. In many cases, the extracted information must be compared to stored information to complete appropriate behaviors.

According to ecological perception theory (Gibson, 1977, 1986), environments consist of five categories of meaningful things: media, substances, surfaces, objects, and animals. Media are long-term constants of the environment. The environment in which humans evolved is made up of air, gravity, and light, which afford breathing, hearing, locomotion, vision, etc. (Gibson, 1986). Animals evolved organs to perceive the affordances of substances, surfaces, objects, and other animals in their media. Substances—what things are made of—differ in rigidity, plasticity, etc. Organisms evolved the perceptual ability to distinguish among substances in order to survive. Surfaces are substance boundaries that create shape and occlusion, which we use to navigate environments. Objects are made up of persisting substances distinguishable by their surfaces. Animals, like objects, have closed body envelopes, but unlike objects, self-generate behavior. Their ever-changing behaviors afford more complex behaviors than objects. Animals provide affordance information *via* their stable features and their transient states that may be more and less motivationally relevant. This means certain types of things about animals may be more likely to be encoded than others.

To illustrate, let us pose a brief example. Suppose you are walking home after dark on a city street, and suddenly you hear screaming. To your left, a man with a bloodied face bursts out of a convenience store waving a gun in the air and immediately runs frantically away, almost knocking you over. According to ecological perception theory, you will directly perceive that the man affords interaction as another self-willed actor. You will also directly perceive that the figure affords threat by brandishing a weapon, screaming, and acting erratically. Thus, you will likely automatically respond to these affordances. However, this situation also presents representation-hungry information because it presents complex parameters to which humans are selectively sensitive: in this case, threat (e.g., blood, weapon, screaming, erratic movements). Adaptively speaking, you should encode the behaviors of the man (how he screamed, where he waved his weapon, etc.), which may prove useful to understand his intentions in the current situation as well as future encounters should you be so unlucky to come across a similar threat in the future. This is directly in line with a motivated attention perspective: threatening behavior is more attended to and more likely encoded and stored for later use.

Non-representation-hungry components also exist within this scenario. You are not likely to encode information about the thief’s shoes or hair color. Nor are you likely to encode exactly what kind of weapon he waved. These stable characteristics are more likely to be reliably available throughout the interaction and in future interactions, so they are less likely to be encoded (Clark, 2008; Lang and Bailey, 2015). You’re also not likely to encode fleeting elements like shadows obscuring his features. Adaptively speaking, you don’t need to remember them to survive, which explains cases of problematic eyewitness testimony. Thus, to predict which motivationally relevant information about animals is more likely to be encoded, we must focus on its representation-hungriness.

## Representation-hungry motivational relevance in animals

Animals provide information to which humans are selectively sensitive: we must perform pattern extraction of their behaviors to behave appropriately ourselves, and this information may not be reliably and consistently available. Which information about animals will be automatically encoded likely depends on current affordances, which depend on both stable features and fleeting states. Stability is defined as the level of permanence in the environment (Gibson, 1986; Lang and Bailey, 2015). Stable elements are typically solid substances that do not change shape easily and thus maintain appearance and allow us to recognize differences between people, places, objects, and other information in the environment (Lang and Bailey, 2015). All animals have stable and unstable aspects; body size, shape and facial features are relatively stable while expressions and gestures are fleeting. Thus, as above, you are more likely to encode the thief’s behavior than hair. However, some stable characteristics about animals may be more likely encoded as well.

Across evolutionary history, interactions with other humans afforded possible opportunities for social interaction as potential mates or friends, but also threats as competitors for scarce resources. Interactions with non-human animals also provided opportunities and threats, and the stable characteristics of these animals pointed to their likelihood as either one or the other (New et al., 2007). A rabbit may afford more opportunities while a wolf may afford more threats. Recognition of these stable characteristics, even in camouflaged circumstances, allow them to be identified and behaviors coordinated appropriately, which provided evolutionary advantage (Altman et al., 2016). Consistent monitoring of behaviors once identified, however, is more likely to shift encoding from stable characteristics to fleeting aspects of their appearances, such as teeth-baring or hair-raising, to understand affordances.

This leads to multiple predictions. First, information about animals should be better encoded than other information. Further, stable, motivationally relevant information may be better encoded than fleeting information about animals as it provides the greatest information about overall affordances. Fleeting, motivationally relevant information about inanimate objects, substances, and surfaces will be better encoded than stable, as these aspects of environmental information are not agentic. These hypotheses were tested in an experiment in which individuals watched audiovisual media and later completed a visual change detection task to examine encoding of fleeting and stable aspects of animals and other non-animate information.

## Methods

### Experimental procedure, design, and stimuli

Participants completed the approximately 45-min protocol in either small groups (in a lab with multiple separated computer cubicles) or individually (in a single-person lab) to maximize data collection.^1^ Upon arrival, informed consent was obtained and participants were seated in appropriate labs. Psychophysiological sensors were affixed for those completing the protocol individually, though these data are not reported here. Participants watched one of two created orders (an order and its reverse to control for order effects) of approximately 30 min of television clips that were combined into relatively naturalistic viewing experiences complete with advertisements (see Table 1). Screenshots were taken approximately every 10 s from this content and used as stimuli in a visual change detection task explained below. After participants viewed this content, they completed a distraction task (e.g., product consumption questionnaire) followed by the change detection task. Participants were debriefed and dismissed. The protocol was approved by the Institutional Review Board.

**TABLE 1:** Stimuli descriptions.

Clip	Description
Allstate commercial	Mayhem character acts as GPS
Addiction cologne commercial	Women fantasize about men wearing cologne
Applebee’s commercial	Diners admire food
At&T commercial	Coverage map comparisons
*Avengers* movie trailer	Avengers defend NYC
Big Ben documentary trailer	History of London’s Big Ben
*Chopped* clip	Contestants prepare food while judges watch
*Contaigion* movie trailer	Man fights with doctor about wife’s death
*CSI Miami* clip	Autopsy discussion
Geico commercial	Robot daycare is less expensive
*How I met your mother* clip	Characters attend birthday party
Integris health commercial	Little boy receives liver transplant
MADD PSA	Police arrest individuals in cars full of alcohol
Anti-tobacco PSA	Mouth cancer shown graphically
Old spice commercial	Man talking to female viewers about their relationships
CBS news clip	Payroll tax legislation coverage
Red lobster commercial	Food being prepared
Rolex commercial	Watch compared to yacht
*Sherlock Holmes* movie trailer	Characters attacked on train
*Spy* vs. *Spy* movie trailer	Spys compete for woman’s affection
Volkswagen commercial	Small boy pretends to be Darth Vader
*Walking dead* trailer	Characters fighting zombies
*Glee* trailer	Characters performing in football halftime show
Citizen watch commercial	Golfer endorses watch

The screenshots utilized in the change detection task were either used as originally viewed or edited using Adobe Photoshop. Half of the targets altered information about an animal. The other half were split between objects, surfaces, and substances. Half of the targets altered stable elements within the scene (i.e., persistent characteristics of animals and objects like color and condition) while the other half changed fleeting elements like emotional expressions and gestures of animals and incidents regarding substances, surfaces such as fire, explosions, or weather (see Table 2 for examples). Foils were unaltered screenshots. Participants viewed target and foil images one at a time, centered, on black screen. DirectRT software presented stimuli in random orders collecting responses and latencies.^2^ Participants were instructed to indicate as quickly as possible whether the image was exactly as seen previously or if anything had changed. They indicated decisions (yes, seen before vs. no, changed) using marked computer keys. If participants did not answer within 3 s, the protocol automatically advanced to the next trial. Data reported here were part of a larger study designed to answer multiple research questions (see also Lang and Bailey, 2015). Participants viewed 160 total trials, 32 unaltered and 128 altered. Stimuli are available from corresponding author upon reasonable request. For this study, we were interested in changes to the most relevant information, that which was relevant to the ongoing video story and to biological motivations. Thus, trials were selected to examine whether stability and animacy influenced encoding within that information category. A 2 (Stability: Stable, Fleeting) × 2 (Animacy: Animals, Objects/Substances/Surfaces) × 5 (trials) within-subjects factorial experiment was employed.

**TABLE 2:** Change detection task examples.

	Animal	Object, substance, surface
Fleeting	Removal of warding off attacker hand/arm gesture during fight in movie trailer	Added explosion visuals (fire, smoke) to additional areas of bridge in movie trailer
Stable	Removal of bandaged wound on person’s face in advertisement	Increased blood stain size on mattress in drama series clip

### Participants

Effect sizes for animacy change detection sensitivity differences were 0.188–0.202 in a similar study (Altman et al., 2016). Specifying a more conservative effect size (0.15) and α = 0.05 in G*Power (Faul et al., 2009), the design requires 84 participants for a 0.95 power estimate. Participants (*N* = 149) were young (*M* = 19.31, *SD* = 1.57, range 18–32) predominantly female (57.7%), undergraduates attending a large public university in the Midwestern United States who received extra credit.

### Data treatment and analysis

To test whether edited images were recognized differently than unedited generally, grand accuracy means for unedited (*M* = 0.624, *SD* = 0.15) and edited (*M* = 0.626, *SD* = 0.19) groups were compared and were not different, *t* (148) = 0.369, *p* = 0.713. Thus, proportion correct data were computed for each cell of the design and submitted to a 2 (Stability: Fleeting, Stable) × 2 (Animacy: Animal, Objects/Substances/Surfaces) repeated-measures ANOVA.

## Results

**FIGURE 1:**
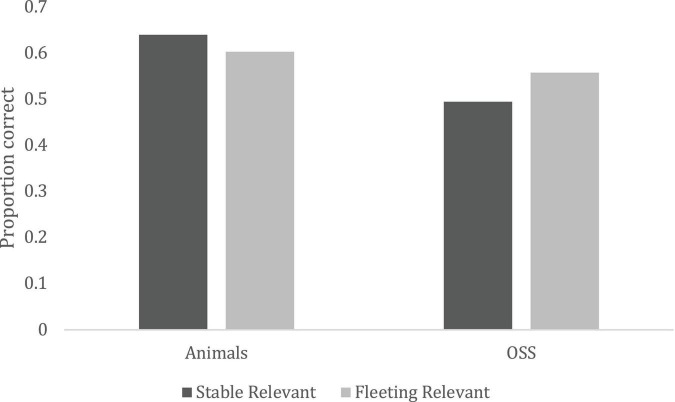
Change detection accuracy as a function of stability (Fleeting vs. Stable), and animacy (Objects, Substances, and Surfaces (OSS) vs. Animal).

Within the category of highly relevant information, changes to animals were best recognized, *F*(1, 148) = 25.46, *p* < 0.0001, η*_*p*_*^2^ = 0.147, but as expected, this interacted with stability, *F*(1, 148) = 7.56, *p* < 0.0001, η*_*p*_*^2^ = 0.049. This interaction is shown in Figure 1. Table 3 contains contrasts. Changes to relevant, stable things about animals (e.g., wounds, teeth, revealing secondary sex characteristics) were significantly better recognized than changes to all non-animate information. Changes to stable and fleeting (e.g., gestures, expressions) information about animals were not significantly different after multiple comparison corrections (*p* > 0.008), but changes to fleeting relevant information about non-animals (e.g., weather, fire) was significantly better recognized (*p* < 0.008) than stable (e.g., blood, food coloration), as predicted.

**TABLE 3:** Stability and animacy interaction contrasts.

Comparison	*t*	*df*	*p*
Fleeting vs. stable animals	1.696	148	0.046
Fleeting vs. stable OSS	2.090	148	0.019
Fleeting OSS vs. fleeting animals	1.839	148	0.034
Stable OSS vs. stable animals	4.998	148	< 0.001*
Fleeting OSS vs. stable animals	3.558	148	< 0.001*
Fleeting animals vs. stable OSS	3.666	148	< 0.001*

*Significant after Bonferonni correction for six tests (α = 0.008).

## Discussion

The motivated attention perspective contends that attention and encoding are biased toward information relevant to biological imperatives. The animate monitoring hypothesis (New et al., 2007) contends the same. Previous research has supported these contentions, but other factors about these information categories may influence their representation-hungriness. Drawing from ecological perception (Gibson, 1986) and situated, extended cognition (Clark and Toribio, 1994; Clark and Chalmers, 1998; Clark, 2008), the approach taken here advocates for additional ecological grounding of attention and encoding processes.

Animals present complex ever-changing affordances. Encoding information about animals, both their stable affordances of threats and opportunities and fleeting affordance shifts, allow us to fine-tune our behaviors. Thus, encoding information about animals is adaptive. Further, though generally predicted that information seen longer should be better encoded, especially perceptual information whose intake may benefit from greater exposure (von Hippel and Hawkins, 1994), a representation-hungry approach about ecologically significant information (vs. word stems and other symbolic information) suggests the opposite. In fact, stable information, from this perspective, would less likely be encoded as it is available for guiding behavior when needed.

These predictions were supported. Highly relevant information about animals, both fleeting and stable, were encoded better than other information types. Further, the encoding of objects, surfaces, and substances was slightly, but significantly, boosted when the elements were fleeting, and thus less likely to be reliably available. This is in line with previous work regarding the animate monitoring hypothesis (New et al., 2007; Altman et al., 2016). Animals, as a category, tend to draw more attention and be better remembered than inanimate environmental information, an effect that could not be accounted for by changes in visual luminance or complexity that may often accompany animateness (New et al., 2007). Further, when an animal was embedded in a camouflaging scene that shared lower-level visual characteristics, animals were still detected more quickly and distracted from monitoring of other inanimate environmental information (Altman et al., 2016). The findings reported here, interpreted considering this previous work, suggest that environmental information with the potential for relevant change is a key predictor of encoding. Animals have consistent potential for relevant change, leading to greater monitoring and encoding in order to interpret ongoing change. Further, inanimate environmental information that presents fleeting changes relevant to ongoing behavior were also better encoded than stable, inanimate information.

Overall, these findings extend well documented attentional influences of motivational relevance and animacy and may add to explanations regarding eyewitness memory issues as well as change blindness—failures to notice large changes in environmental stimuli—(see Simons and Rensink, 2005). It could be that information not noticed may be stable and not encoded initially, and, therefore, not able to be well recognized later. This may be related to “gist” representations within fuzzy trace theory (Reyna and Brainerd, 1995), where individuals encode semantic information rather than “verbatim” perceptual representations that may be more relevant to discussions of representation-hungriness. Perceptual environmental features are more likely to be representation-hungry, or encoded for later use, when it relates to threat and opportunity and presents the potential for relevant change in these factors. Animals, as a category, afford the most threat and opportunity, and can shift these biologically relevant potentials from moment-to-moment, but other environmental information also fit these criteria and may be similarly representation-hungry as a result. Thus, as previously suggested, human cognitive architecture may have evolved mechanisms that allocate encoding resources to these types of stimuli (New et al., 2007).

Multiple limitations should be considered. The laboratory environment may have created more goal-oriented focus that yielded these results. Further, stimuli were not selected by participants. In real-life, individuals control their media choices, creating initial conditions that may change encoding processes. Also of note, this study did not control for recollections of previous content exposure. As repeated exposure is likely to increase encoding, future research should also investigate whether these factors influence reported outcomes.

Overall, this study adds to motivational relevance explications and suggests avenues for extension of functional explanations of cognition.

## Data availability statement

The raw data supporting the conclusions of this article will be made available by the authors, without undue reservation.

## Ethics statement

The studies involving human participants were reviewed and approved by the Indiana University Institutional Review Board. The patients/participants provided their written informed consent to participate in this study.

## Author contributions

RB: design, data collection, analysis, and writing. AL: design and writing. Both authors contributed to the article and approved the submitted version.

## Footnotes

1 Both labs offered privacy and limited distractions. No effect of lab was found in overall analysis. This factor was omitted in further analysis.

2 Latency data are not reported here as they are not central to the main outcome of interest.
